# Delayed orthostatic hypotension in Parkinson’s disease

**DOI:** 10.1038/s41531-021-00181-y

**Published:** 2021-04-14

**Authors:** Sang-Won Yoo, Joong-Seok Kim, Ji-Yeon Yoo, Eunkyeong Yun, Uicheul Yoon, Na-Young Shin, Kwang-Soo Lee

**Affiliations:** 1 Department of Neurology, College of Medicine, The Catholic University of Korea, Seoul, Republic of Korea; 2 Department of Biomedical Engineering, College of Health and Medical Science, Catholic University of Daegu, Gyeongbuk, Republic of Korea; 3 Department of Radiology, College of Medicine, The Catholic University of Korea, Seoul, Republic of Korea

**Keywords:** Neurological manifestations, Parkinson's disease

## Abstract

Orthostatic hypotension (OH) is relatively common in the early stage of Parkinson’s disease (PD). It is divided into delayed OH and classical OH. Classical OH in PD has been investigated widely, however, the clinical implications of delayed OH in PD have seldom been studied. The purpose of this study is to characterize delayed OH in PD. A total of 285 patients with early drug-naïve PD were enrolled and divided into three groups according to orthostatic change: no-OH, delayed OH, and classical OH. The disease severity in terms of motor, non-motor, and cognitive functions was assessed. The cortical thickness of 82 patients was analyzed with brain magnetic resonance imaging. The differences among groups and linear tendency in the order of no-OH, delayed OH, and classical OH were investigated. Seventy-seven patients were re-evaluated. Initial and follow-up evaluations were explored to discern any temporal effects of orthostasis on disease severity. Sixty-four (22.5%) patients were defined as having delayed OH and 117 (41.1%) had classical OH. Between-group comparisons revealed that classical OH had the worst outcomes in motor, non-motor, cognitive, and cortical thickness, compared to the other groups. No-OH and delayed OH did not differ significantly. Linear trends across the pre-ordered OH subtypes found that clinical parameters worsened along with the orthostatic challenge. Clinical scales deteriorated and the linear gradient was maintained during the follow-up period. This study suggests that delayed OH is a mild form of classical OH in PD. PD with delayed OH has milder disease severity and progression.

## Introduction

Dysautonomia is a well-known non-motor feature that is discovered in the prodrome of Parkinson’s disease (PD)^1,2^. Orthostatic hypotension (OH) is found in early drug-naïve PD^3^, and its presence is consistently associated with worse outcomes^4–7^.

OH is clinically divided into classical OH and delayed OH^8,9^. Classical OH is conventionally defined as sustained decrease in systolic blood pressure (SBP) ≥ 20 mmHg and/or diastolic blood pressure (DBP) 10 mmHg within 3 min of standing, and delayed OH is considered when the progressive blood pressure drop surpasses the margin of change after 3 min. The clinical implications of delayed OH are suggested to be a non-benign, mild, or early form of sympathetic adrenergic dysfunction^10^. Although its significance was seldom studied in populations with PD, a longitudinal follow-up study revealed some individuals with delayed OH progressed to classical OH and developed α-synucleinopathy^11^.

The assumption of present study was that delayed OH is a mild form of neurogenic OH in early PD, representing milder disease severity. Patients were sub-grouped into a hypothesis-driven ordinal scale. The expected outcomes were between-group differences and a linear gradient across the subtypes, and results that were maintained with time. This would prove that patients with delayed OH have different motor and non-motor phenotypes and a distinct prognosis from other groups.

## Results

### Baseline characteristics

**Fig. 1:**
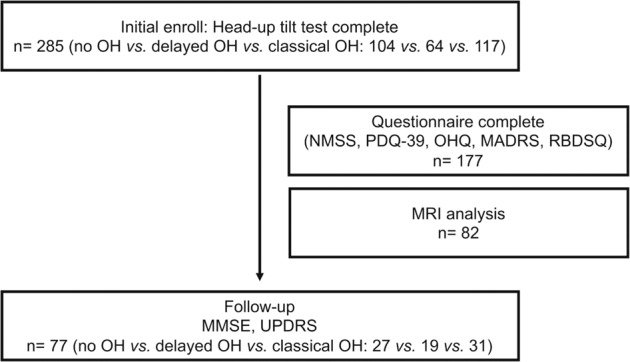
Enrolled patients study flow. *NMSS* Non-Motor Symptoms Scale, *PDQ-39* Parkinson’s Disease Quality of Life-39, *OHQ* Orthostatic Hypotension Questionnaire, *MADRS* Montgomery-Asberg Depression Rating Scale, *RBDSQ* REM Sleep Behavior Disorder Screening Questionnaire, *UPDRS* Unified Parkinson’s Disease Rating Scale, *MMSE* Mini-Mental Status Examination.

The flow of participants is presented in Fig. 1 and the baseline demographics of PD patients are summarized in Table 1. The mean age was 69.6 ± 9.3 years old and 132 (46.3%) were female. The median duration of disease was 1 (1.0) year (interquartile range, IQR). The mean total sum of Unified Parkinson’s Disease Rating Scale (UPDRS) was 23.3 ± 12.6 with a median modified Hoehn and Yahr (H&Y) of 2.0 (IQR, 1.0). The mean score of Mini-Mental Status Examination (MMSE) was 27.0 ± 2.8 and the median Clinical Dementia Rating (CDR) was 0.5 (IQR, 0). Sixty-four patients (22.5%) were defined to have delayed OH and 117 (41.1%) had classical OH. The mean uptake of delayed heart-to-mediastinum (H/M) ratio was 1.55 ± 0.37. Classical OH were older than other groups.

**Table 1:** Clinical characteristics.

		PD (*n* = 285)	Orthostatic challenge				*P*-value	Post hoc analysis
			no-OH (*n* = 104)	Delayed OH (*n* = 64)	Classical OH (*n* = 117)	Test statistics (*F* or *χ*^2^)		
Age, year	Mean ± SD	69.6 ± 9.3	67.7 ± 10.7	67.3 ± 8.0	72.6 ± 7.7	12.5	<0.001	*a* < c***, *b* < c***
Sex, female	*n* (%)	132 (46.3)	55 (52.9)	35 (54.7)	42 (35.9)	8.72	0.013	–
Body mass index (Kg/m^2^)	Mean ± SD	23.8 ± 3.0	24.1 ± 3.0	24.2 ± 3.0	23.5 ± 2.9	1.64	0.195	–
Disease duration, years	Median (IQR)	1.0 (1.0)	1.0 (1.0)	0.6 (0.6)	1.0 (1.5)	6.72	0.035	*a* > b*
Education, years	Mean ± SD	11.1 ± 4.7	11.2 ± 4.9	10.8 ± 4.5	11.1 ± 4.6	0.19	0.829	–
Hypertension	*n* (%)	125 (43.9)	46 (44.2)	27 (42.2)	52 (44.4)	0.09	0.954	–
CCBs^d^	*n* (%)	74 (26.0)	25 (24.0)	17 (26.6)	32 (27.4)	0.33	0.848	–
ARBs^d^	*n* (%)	76 (26.7)	27 (26.0)	16 (25.0)	33 (28.2)	0.26	0.879	–
β-blockers^d^	*n* (%)	16 (5.6)	4 (3.8)	4 (6.3)	8 (6.8)	0.99	0.609	–
Diuretics^d^	*n* (%)	23 (8.1)	6 (5.8)	4 (6.3)	13 (11.1)	2.49	0.288	–
Diabetes mellitus	*n* (%)	43 (15.1)	11 (10.6)	13 (20.3)	19 (16.2)	3.14	0.208	–
Dyslipidemia	*n* (%)	81 (28.4)	21 (20.2)	22 (34.4)	38 (32.5)	5.52	0.063	–
Stroke	*n* (%)	5 (1.8)	4 (3.8)	0 (0.0)	1 (0.9)	4.33	0.115	–
Coronary artery disease	*n* (%)	23 (8.1)	7 (6.7)	7 (10.9)	9 (7.7)	0.98	0.612	–
Current smoker	*n* (%)	12 (4.2)	3 (2.9)	2 (3.1)	7 (6.0)	1.55	0.460	–
MMSE	Mean ± SD	27.0 ± 2.8	27.4 ± 2.3	27.2 ± 2.9	26.4 ± 3.0	3.79	0.024	*a* > c*
CDR	Median (IQR)	0.5 (0.0)	0.5 (0.0)	0.5 (0.0)	0.5 (0.0)	0.16	0.924	–
Modified H&Y	Median (IQR)	2.0 (1.0)	2.0 (1.0)	2.0 (1.0)	2.0 (0.5)	9.86	0.007	*a* < c**

Analysis of variance was used for continuous variables and the *χ*^2^ test for categorical variables. Non-normally distributed variables were analyzed by the Kruskal–Wallis test. Pairwise multiple comparisons were adjusted by Tukey’s HSD, Games-Howell or Dwass-Steel-Critchlow-Fligner tests, when appropriate.

*PD* Parkinson’s disease, *OH* orthostatic hypotension, *CCB* calcium channel blocker, *ARB* angiotensin II receptor blocker, *MMSE* Mini-Mental Status Examination, *CDR* Clinical Dementia Rating, *H&Y* Hoehn and Yahr, *SD* standard deviation, *IQR* interquartile range.

*p-value < 0.05, **p-value < 0.01, ***p-value < 0.001

^a^no-OH, ^b^Delayed OH, ^c^Classical OH, ^d^Some patients were prescribed in combinations.

### Comparisons across orthostatic hypotension groups

Between-group comparisons, adjusted for age, sex, and disease duration were analyzed and encapsulated in Table 2 and Supplementary Table 1. Classical OH scored higher in UPDRS Part II (no-OH vs. delayed OH *vs*. classical OH: 5.2 ± 0.4 vs. 5.1 ± 0.5 vs. 6.8 ± 0.4; *p* = 0.005; *a* = *b* < *c*), Part III (no-OH *vs*. delayed OH *vs*. classical OH: 14.9 ± 0.8 vs. 13.9 ± 1.1 vs 17.9 ± 0.8; *p* = 0.008; *a* = *b* < *c*), and UPDRS total score (no-OH *vs*. delayed OH *vs*. classical OH: 21.4 ± 1.1 vs. 20.7 ± 1.5 vs. 26.4 ± 1.1; *p* = 0.002; *a* < *c*). The classical OH group also had higher supine systolic blood pressure than the no-OH group. Of 285 PD, 154 (54.0%) had nocturnal hypertension and 138 (48.4%) were non-dippers. UPDRS scores, supine blood pressure (BP), the proportions of nocturnal hypertension, and non-dipper all showed an ascending linear trend across the OH groups.

**Table 2:** Between-group comparisons across orthostatic hypotension.

		no-OH (*n* = 104)	Delayed OH (*n* = 64)	Classical OH (*n* = 117)	Test statistics (*F* or *χ*^2^)	*P*-value	Post hoc analysis	*P* for linear trend
UPDRS, total	Mean ± SEM	21.4 ± 1.1	20.7 ± 1.5	26.4 ± 1.1	6.44	0.002	*a* < c**, *b* < c**	0.002
UPDRS Part I	Mean ± SEM	1.3 ± 0.1	1.7 ± 0.2	1.7 ± 0.1	2.67	0.071		0.030
UPDRS Part II	Mean ± SEM	5.2 ± 0.4	5.1 ± 0.5	6.8 ± 0.4	5.44	0.005	*a* < c**, *b* < c*	0.003
UPDRS Part III	Mean ± SEM	14.9 ± 0.8	13.9 ± 1.1	17.9 ± 0.8	4.96	0.008	*a* < c*, *b* < c*	0.013
Supine SBP	Mean ± SEM	126.4 ± 1.6	128.4 ± 2.0	134.1 ± 1.6	5.97	0.003	*a* < c**	0.001
Supine DBP	Mean ± SEM	73.9 ± 0.9	74.8 ± 1.2	76.3 ± 0.9	1.51	0.222		0.085
Orthostatic ∆SBP	Mean ± SEM	6.7 ± 0.8	14.7 ± 1.0	27.6 ± 0.8	174.87	<0.001	*a* < *b* < *c* ***	<0.001
Orthostatic ∆DBP	Mean ± SEM	0.9 ± 0.5	4.4 ± 0.7	12.3 ± 0.5	126.86	<0.001	*a* < *b* < *c* ***	<0.001
Nocturnal hypertension	*n* (%)	46 (44.2)	33 (51.6)	75 (64.1)	8.96	0.011	–	0.003
Non-dipper	*n* (%)	38 (36.5)	31 (48.4)	69 (59.0)	11.10	0.004	–	0.001
Early H/M ratio	Mean ± SEM	1.68 ± 0.03	1.53 ± 0.04	1.51 ± 0.03	9.87	<0.001	*a* > b**, *a* > c***	<0.001
Delayed H/M ratio	Mean ± SEM	1.67 ± 0.04	1.50 ± 0.05	1.46 ± 0.03	10.27	<0.001	*a* > b**, *a* > c***	<0.001
Questionnaire		No-OH^a^ (*n* = 68)	Delayed OH^b^ (*n* = 38)	Classical OH^c^ (*n* = 71)	–	–	–	–
NMSS total	Mean ± SEM	30.2 ± 4.5	33.9 ± 6.1	47.1 ± 4.6	3.53	0.032	*a* < c*	0.011
PDQ39 SI	Mean ± SEM	13.1 ± 1.4	12.0 ± 1.9	15.8 ± 1.4	1.46	0.234	–	0.194
OHQ Part I	Mean ± SEM	5.8 ± 1.0	6.0 ± 1.4	8.5 ± 1.0	1.96	0.144	–	0.065
OHQ Part II	Mean ± SEM	4.7 ± 1.1	4.8 ± 1.5	8.7 ± 1.1	3.74	0.026	*a* < c*	0.013
MADRS sum	Mean ± SEM	5.1 ± 0.8	7.1 ± 1.1	7.4 ± 0.8	2.25	0.109		0.048
RBDSQ	Mean ± SEM	2.5 ± 0.3	3.6 ± 0.4	3.8 ± 0.3	3.79	0.025	*a* < c*	0.011

Values are mean ± standard error of the mean (SEM), unless otherwise indicated.

Blood pressures were measured as mmHg units.

Analysis of covariance, adjusted for age, sex, and disease duration, was applied to compare between-group differences. Pairwise multiple comparisons were adjusted by Tukey’s HSD. The *χ*2 test was done for categorical variables. Polynomial contrasts or Cochran-Armitage tests were performed, when appropriate, to appreciate the linear trend.

*OH* orthostatic hypotension, *UPDRS* Unified Parkinson’s Disease Rating Scale, *SBP* systolic blood pressure, *DBP* diastolic blood pressure, *H/M* heart-to-mediastinum, *NMSS* Non-Motor Symptoms Scale, *PDQ39 SI* Parkinson’s Disease Quality of Life-39 summary index, *OHQ* Orthostatic Hypotension Questionnaire, *MADRS* Montgomery-Asberg depression rating scale, *RBDSQ* REM Sleep Behavior Disorder Screening Questionnaire.

*p-value < 0.05, **p-value < 0.01, ***p-value < 0.001.

^a^no-OHX, ^b^Delayed OH, ^c^Classical OH.

Classical OH had higher Non-Motor Symptoms Scale (NMSS) scores (no-OH vs. classical OH: 30.2 ± 4.5 vs. 47.1 ± 4.6; *p* = 0.032), OHQ Part II (no-OH vs. classical OH: 4.7 ± 1.1 vs. 8.7 ± 1.1; *p* = 0.026), and Rapid-eye-movement Sleep Behavior Disorder Screening Questionnaire (RBDSQ; no-OH vs. classical OH: 2.5 ± 0.3 vs. 3.8 ± 0.3; *p* = 0.025) than no-OH. A positive linear trend was found across the groups in the NMSS, Orthostatic Hypotension Questionnaire (OHQ) Part II, Montgomery-Asberg Depression Rating Scale (MADRS), and RBDSQ questionnaires.

Cortical thickness comparison also depicted a similar pattern of between-group differences, when controlled for age, sex, disease duration, and MMSE. The whole cerebral cortex (no-OH vs. classical OH: 3.27 ± 0.02 vs. 3.15 ± 0.03; *p* = 0.002), in particular the frontal and parietal lobes (no-OH vs. classical OH: 3.17 ± 0.02 vs. 3.05 ± 0.02, *p* = 0.001; 2.98 ± 0.03 vs. 2.79 ± 0.05, *p* = 0.007; respectively), manifested thinner thickness in patients with classical OH group compared to no-OH. There was a negative linear tendency in the general cortex, except the left temporal and occipital cortex, across the OH groups (Table 3).

**Table 3:** Between-group differences of cortical thickness across orthostatic hypotension.

Cortical areas		no-OH (*n* = 36)	Delayed OH (*n* = 25)	Classical OH (*n* = 21)	Test statistics (*F*)	*P*-value	Post hoc analysis	*P* for linear trend
Frontal	Mean ± SEM	3.17 ± 0.02	3.14 ± 0.02	3.05 ± 0.02	7.96	0.001	*a* > c***, *b* > c*	<0.001
Right	Mean ± SEM	3.18 ± 0.02	3.15 ± 0.02	3.06 ± 0.03	7.49	0.001	*a* > c***, *b* > c*	<0.001
Left	Mean ± SEM	3.16 ± 0.02	3.12 ± 0.02	3.05 ± 0.02	7.00	0.002	*a* > c**	<0.001
Parietal	Mean ± SEM	2.98 ± 0.03	2.88 ± 0.04	2.79 ± 0.05	5.37	0.007	*a* > c**	0.002
Right	Mean ± SEM	2.96 ± 0.03	2.88 ± 0.04	2.79 ± 0.05	4.68	0.012	*a* > c**	0.004
Left	Mean ± SEM	3.00 ± 0.04	2.88 ± 0.05	2.80 ± 0.05	5.11	0.008	*a* > c**	0.003
Temporal	Mean ± SEM	3.50 ± 0.03	3.44 ± 0.03	3.38 ± 0.04	3.09	0.051	–	0.018
Right	Mean ± SEM	3.55 ± 0.03	3.49 ± 0.04	3.41 ± 0.04	3.76	0.028	*a* > c*	0.008
Left	Mean ± SEM	3.43 ± 0.03	3.38 ± 0.03	3.35 ± 0.04	1.76	0.179	–	0.079
Occipital	Mean ± SEM	3.38 ± 0.04	3.26 ± 0.05	3.26 ± 0.05	2.62	0.079	–	0.072
Right	Mean ± SEM	3.47 ± 0.05	3.29 ± 0.06	3.32 ± 0.06	3.21	0.046	–	0.074
Left	Mean ± SEM	3.30 ± 0.04	3.23 ± 0.05	3.20 ± 0.05	1.18	0.313	–	0.168
Whole	Mean ± SEM	3.27 ± 0.02	3.22 ± 0.02	3.15 ± 0.03	6.62	0.002	*a* > c**	0.001
Right	Mean ± SEM	3.31 ± 0.02	3.25 ± 0.03	3.17 ± 0.03	6.61	0.002	*a* > c**	0.001
Left	Mean ± SEM	3.24 ± 0.02	3.19 ± 0.02	3.13 ± 0.03	5.24	0.007	*a* > c**	0.002

Analysis of covariance, adjusted for age, sex, disease duration, and Mini-Mental Status Examination, was applied to compare between-group differences. Pairwise multiple comparisons were adjusted by Tukey’s HSD. Polynomial contrasts were performed, when appropriate, to appreciate the linear trend.

*OH* orthostatic hypotension, *SEM* standard error of the mean.

*p-value < 0.05, **p-value < 0.01, ***p-value < 0.001.

^a^no-OH, ^b^Delayed OH, ^c^Classical OH.

### Longitudinal influences of orthostatic hypotension groups

**Fig. 2:**
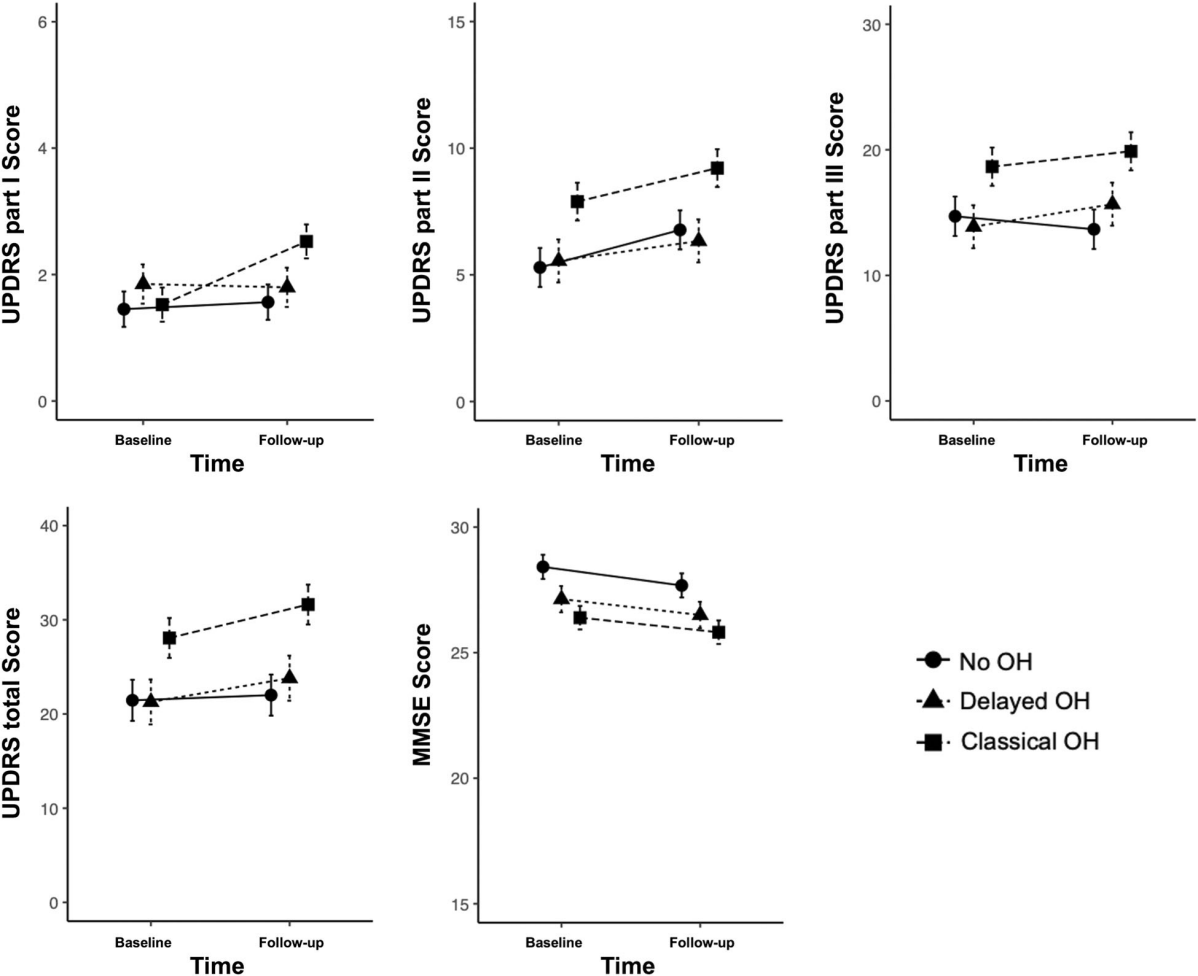
Temporal progression of cognitive and motor severities across the groups. Error bars: standard error of the mean. *UPDRS* Unified Parkinson’s Disease Rating Scale, *MMSE* Mini-Mental Status Examination, *OH* orthostatic hypotension.

Seventy-seven PD patients were followed for a mean period of 24.2 ± 5.3 months (no-OH vs. delayed OH vs. classical OH: 23.3 ± 5.1 vs. 23.0 ± 4.8 vs. 25.7 ± 5.5; *p* = 0.117) and the longitudinal influences of the orthostatic BP drop are presented in Table 4 and Fig. 2. In this sub-analysis, age, disease duration and levodopa equivalent daily dose (LEDD) of the last trace point did not differ across the groups (age: *p* = 0.065; disease duration: *p* = 0.531; LEDD: *p* = 0.137). UPDRS Part II and MMSE score demonstrated progression during the follow-up period (time effects: *p* = 0.019, *p* = 0.008; respectively) and classical OH had worse outcomes on these scales (group effects: *p* = 0.006, *p* = 0.006; *a* = *b* < *c*, *a* > *c*; respectively). UPDRS total sum and Part III did not display any impairment during the follow-up (time effects: *p* = 0.075, *p* = 0.440; respectively), but maintained between-group differences (group effects: *p* = 0.004, *p* = 0.016; respectively). There were no interactions between time and group effects among the analyzed scales. When dopaminergic treatment effects were adjusted, UPDRS scores and MMSE did not worsen significantly over the follow-up period (time effect: *p* > 0.05). Classical OH similarly maintained its worsening trend on UPDRS total sum, Part II, Part III and MMSE scores (group effects: *p* = 0.012, *p* = 0.023, *p* = 0.034, *p* = 0.006; interaction: *p* = 0.574, *p* = 0.872, *p* = 0.346; *p* = 0.874; Supplementary Table 2 and Supplementary Fig. 1).

**Table 4:** Influence of initial orthostatic test on cognitive and motor symptoms progression.

	no-OH (*n* = 27)		Delayed OH (*n* = 19)		Classical OH (*n* = 31)								
	Baseline	Follow-up	Baseline	Follow-up	Baseline	Follow-up					*P*-value		
Age^d^	66.9 ± 11.3	68.9 ± 11.3	67.4 ± 5.2	69.3 ± 5.2	71.4 ± 8.1	73.6 ± 8.2					0.065		
Disease duration^d^ (median, IQR)	1.0 (1.0)	2.9 (1.0)	0.7 (0.5)	2.7 (0.6)	1.0 (1.5)	3.1 (1.2)					0.531		
Time to follow-up^d^	–	23.3 ± 5.1	–	23.0 ± 4.8	–	25.7 ± 5.5					0.117		
LEDD^d^	–	426.5 ± 165.5	–	409.2 ± 120.0	–	528.2 ± 287.3					0.137		
							**Time effect**		**Group effect**		**Post hoc analysis**	**Interaction**	
							***F***	***P***	***F***	***P***		***F***	***P***
UPDRS, total	21.0 ± 9.4	21.5 ± 10.3	20.8 ± 5.9	23.3 ± 10.4	27.6 ± 12.0	31.1 ± 14.6	3.25	0.075	5.85	0.004	*a* < c**, *b* < c*	0.59	0.556
UPDRS Part I	1.4 ± 1.1	1.6 ± 1.6	1.8 ± 1.6	1.8 ± 1.1	1.5 ± 1.4	2.5 ± 1.6	3.07	0.084	1.49	0.231	–	2.88	0.063
UPDRS Part II	5.1 ± 2.8	6.6 ± 3.9	5.4 ± 2.4	6.2 ± 4.0	7.7 ± 4.0	9.0 ± 5.3	5.75	0.019	5.58	0.006	*a* < c*, *b* < c*	0.15	0.858
UPDRS Part III	14.4 ± 7.4	13.4 ± 7.5	13.6 ± 3.7	15.4 ± 7.1	18.4 ± 8.7	19.6 ± 10.2	0.59	0.440	4.34	0.016	*a* < c*	1.03	0.362
MMSE	28.4 ± 1.4	27.7 ± 2.0	27.2 ± 2.0	26.5 ± 2.6	26.4 ± 2.6	25.8 ± 3.3	7.49	0.008	5.55	0.006	*a* > c**	0.05	0.956
CDR (median, IQR)	0.5 (0.0)	0.5 (0.0)	0.5 (0.0)	0.5 (0.0)	0.5 (0.5)	0.5 (0.0)	–	–	–	–	–	–	–

Repeated measures analysis of variance was applied to discern within and between-group differences. Pairwise multiple comparisons were adjusted by Tukey’s HSD.

Values are mean ± standard deviation unless otherwise indicated. Time, group, and time × group interaction effect values are *P* values.

*OH* orthostatic hypotension, *LEDD* levodopa equivalent daily dose, *UPDRS* Unified Parkinson’s Disease Rating Scale, *MMSE* Mini-Mental Status Examination, *CDR* Clinical dementia rating, *IQR* interquartile range.

*p-value < 0.05, **p-value < 0.01, ***p-value < 0.001.

^a^no-OH, ^b^Delayed OH, ^c^Classical OH, ^d^Between-group differences of age, disease duration, time interval and amount of levodopa dose were not found by analysis of variance or Kruskal-Wallis test, when appropriate.

## Discussion

In this study, ordinal subtypes of OH (delayed OH *vs*. classical OH) were compared to investigate the nature of OH in early PD. Group with classical OH manifested more severe clinical scales and biomarkers than other groups. However, no-OH and delayed OH groups did not differ significantly. The disparities had a linear gradient pattern across the OH spectrum. These findings were sustained during the longitudinal follow-up.

The population of this study was in the early stage of disease with non-demented mild PD. The similar clinical status reflects a relatively homogeneous pathophysiologic stage confined to the brainstem according to ‘bottom-up’ theory^12,13^. PD patients with classical OH were older than other groups, and the prevalence of classical OH was similar when compared to previous studies that estimated it in a range of 14–54%^3^.

The prevalence of delayed OH was also comparable^11^, but with a different clinical context. Gibbons and Freeman explored the natural history of delayed OH in a 10-year follow-up study and observed that about half progressed to classical OH. Among the subjects with delayed OH in initial testing who progressed to classical OH, >50% developed α-synucleinopathy. This study was not designed to investigate delayed OH in PD; therefore, it was difficult to infer any clinical significance in PD population. Our research is of value that it studied delayed OH in PD.

Motor related (dopaminergic) functions, including daily activities, were preferentially more affected in PD with classical OH when the covariates were controlled. Motor features of daily activities worsened over a 2-year period, in line with a previous study^14^. The differences among groups and aggravating linear trend of disease severity across groups were maintained throughout the follow-up period. The potential causes of the motor disparities could be the result of severer underlying disease burden and/or be attributed to fatigue or end-organ damage by blood pressure instability^15,16^.

Non-motor features were found to be more severe in PD with classical OH. Worse non-motor features could have affected adverse motor outcomes^17^. The participants showed increasing non-motor severity across the OH groups. The positive association of nondopaminergic parameters with ordinal OH further strengthens the argument that delayed OH is a mild form of neurogenic OH in early PD. The cognition of all three groups worsened with time. PD with classical OH progressed more aggressively while the worsening of no-OH and delayed OH were not disparate. This result is consistent with a previous study where OH is reported to impact cognitive decline^5,18^.

After adjusting for dopaminergic therapy, the cognitive and motor severities did not demonstrate significant worsening, regardless of the OH types. This was anticipatory because early non-demented PD (motor and cognitive aspects) responded to dopaminergic replacement^19^. However, classical OH had worse overall severities than the other groups, and its unfavorable outcomes were sustained during the follow-up period, independent of dopaminergic treatment. The observed baseline between-group differences and worsening tendencies across the OH spectrum during the follow-up period argues that different levels of orthostatic challenge may affect distinctive clinical progressions.

Association between OH and cognitive impairment has been discussed previously. In this cohort of early de novo PD, noradrenergic deficiency by locus coeruleus impairment and vascular theory of cerebral hypoperfusion could have attributed to the more rapid decline of cognition in classical OH^20,21^. Cortical thinning could also be a cause of such a finding.

Biomarkers of the cardiovascular system and cortical thickness depicted similar patterns across the groups as the clinical parameters. The frequency of disrupted circadian blood pressures, assessed by 24-hour ambulatory blood pressure monitoring, increased with the planned order of OH types. Patients with OH (both classical or delayed) were more aggressively deprived of cardiac sympathetic innervation than those with no-OH. Supine blood pressure tended to increase across the OH spectrum. The worsening gradient across the groups represented more damaged arterial baroreflex abnormalities^1^. Anterior dominant cortical thinning was worst in the classical OH. This was replicated in a previous study with hypotensive insults as a causative mechanism^22–25^. The cortex tended to become thinner across the OH groups which may result from different levels of hypotensive insults.

The strength of this study was that a large number of early drug-naïve PD were enrolled with extensive assessments encompassing non-motor and motor features. Other studies with comparable early PD involved small populations^26,27^. Only recently, a large scale prospective cohort was published however this performed limited evaluations and provided limited information^3^. PD is an age-dependent neurodegenerative disease in which clinical parameters are affected by aging. In this study, baseline characteristics with demonstrated differences were re-assessed with an adjustment of covariates, unlike in previous studies^3^. This study has several limitations. First, the enrolled patients did not go through every measurement due to the condition of each patient, and they were excluded from sub-analyses. In addition, many patients were lost during the follow-up. The large drop-outs are the major weakness because this would inevitably deepen selection bias. However, the study population included mild non-demented de novo PD to ensure reasonably homogenous pathologic stages and was followed for 2-year period on average. Its homogeneity of mild disease severity and relatively short follow-up duration may mitigate the bias that only the fitter patients could have been investigated. The large drop-outs could reduce the statistical power to discern group differences. On the contrary, it supports that achieved differences overcame the conservative null hypothesis produced by diminished statistical power; however, careful consideration is required in the interpretation since the drop-outs could also bias the alternative hypothesis. Second, autonomic evaluations were not comprehensive. Further evaluations of parasympathetic outflows could be of benefit in elucidating the pathophysiology of delayed OH. Third, patients had comorbid diseases, such as hypertension and diabetes mellitus, which contribute to autonomic disturbances. In addition, many subjects with comorbid diseases were on medications. We did not analyze the interactive influence of systemic diseases or drugs that might play a pathophysiologic role in autonomic failure. Finally, the follow-up duration was too short for definite conclusiveness. Large prolonged prospective studies with comprehensive evaluations of clinical aspects and biomarkers are required to further describe delayed OH.

In summary, not only the pattern of between-group differences but also the linear gradient across the ordinal subtypes of OH, and the maintenance of such traits with time suggest that delayed OH is a mild form of classical neurogenic OH in early PD, associated with less severe clinical burden and deterioration. This may facilitate patient selections in future neuroprotective studies.

## Methods

### Participants

This longitudinal study was approved by the Institutional Review at Seoul St. Mary’s Hospital, and all subjects provided written informed consent to participate. All experiments were performed in accordance with relevant guidelines and regulations. The study was registered (Identification Number: KCT0005552) in the Clinical Research Information Service (CRIS; http://cris.nih.go.kr), which is an online clinical trial registration system established by the Korea Centers for Disease Control and Prevention (KCDC) with support from the Korea Ministry of Health and Welfare (KMOHW) and embodied as a part of the Primary Registries in the World Health Organization (WHO) Registry Network.

Two hundred eighty-five drug-naïve patients newly diagnosed with PD between October 2014 and December 2019 were enrolled. The diagnosis of PD was based on the UK Parkinson’s Disease Society Brain Bank clinical diagnostic criteria^28^, and its diagnosis was substantiated by positron emission tomography imaging studies using ^18^F-N-(3-fluoropropyl)-2beta-carbon ethoxy-3beta-(4-iodophenyl) nortropane and ^123^I-metaiodobenzylguanidine (^123^I-MIBG) scintigraphy^29,30^. Baseline demographics such as age, sex, body mass index (BMI), disease duration, education, smoking status, and history of hypertension, diabetes mellitus, dyslipidemia, stroke, and coronary artery disease were investigated. Disease severity was evaluated with the UPDRS and modified H&Y stage. Global cognition was assessed by MMSE and CDR.

Patients with any of the following indications were excluded from the initial enrollment: (1) any symptoms or signs of atypical and/or secondary parkinsonism, (2) documentation of atrial fibrillation during head-up tilt electrocardiographic monitoring, (3) history of diabetic neuropathy, (4) history of peripheral arterial disease, (5) history of symptomatic stroke that might affect cognition and general performance, (6) taking medications such as tricyclic antidepressant and alpha-adrenergic antagonists that influence orthostatic challenge, (7) family history of dementia, and (8) clinical suspicion of dementia (CDR ≥ 1)^31^.

The global cognitive efficiency and disease severity of 77 patients with PD were re-evaluated by using MMSE and UPDRS, respectively (Fig. 1).

### Questionnaires

Questionnaires were completed by 177 patients. Non-motor features, mood, quality of life, parasomnia, and symptoms related to orthostatic challenge were evaluated with the NMSS, MADRS, Parkinson’s Disease Quality of Life-39 (PDQ-39), RBDSQ and OHQ, respectively^32–36^. The sums of each questionnaire were used for analyses.

### Head-up tilt test

All patients were at a full resting state before the exam. Continuous electrocardiograph leads and non-invasive blood pressure monitoring equipment were applied to the patients (YM6000, Mediana Tech, Redmond, WA, USA). A supine position was maintained for 20 min before tilting to 60° (Enraf-Nonius, Rotterdam, The Netherlands). While in the supine position, blood pressure and heart rate were measured every 5 min for 20 min. At the 60° position, blood pressure and heart rate were measured at 0, 3, 5, 10, 15, and 20 min. For analyses, the lowest tilt values for BP were chosen from 0 to 3 min for classical OH, and from 5 to 20 min for delayed OH. The first supine blood pressure (at 0 min) was excluded, and maximal supine systolic and diastolic blood pressures were selected among the measurements at 5, 10, 15, and 20 min. The lowest systolic and diastolic values at 0 or 3 min during the tilted position were chosen. The orthostatic blood pressure changes of systole (*Δ*SBP) and diastole (*Δ*DBP) were calculated. Patients were categorized as having classical OH or delayed OH if *Δ*SBP and/or *Δ*DBP ≥ 20/10 mmHg within 3 min or when the BP drops occurred after 5 min^8^. Cases that satisfied both classical OH and delayed OH criteria were categorized as classical OH.

### Twenty-four-hour ambulatory blood pressure monitoring

Automated 24-hour blood pressure equipment (Mobil-O-Graph NG, IEM, Stolberg, Germany) was used to measure daytime and nighttime blood pressures from the upper arm. Blood pressures were recorded at 15-minute intervals throughout the day and 30-min intervals at night. Daytime was defined as a period between 8:00 a.m. and 23:59 p.m., and nighttime was from 00:00 a.m. to 07:59 a.m. Nocturnal hypertension was defined as increased absolute values of nighttime systolic and/or diastolic BP ≥ 120/70 mmHg^37^. Patients were classified as non-dipper if the ratio of night/day systolic and/or diastolic BP ≥ 1^38^.

### 123I-metaiodobenzylguanidine scintigraphy

^123^I-MIBG scintigraphy was performed using a dual-head camera equipped with a low-energy high-resolution collimator (Siemens), and data were collected at 30-min (early) and 2-h (delayed) time points after a 111 MBq ^123^I-MIBG injection. A static image was obtained with a 128 × 128 matrix. Regions of interest were manually drawn around the heart and mediastinum. Tracer uptake was measured within each region of interest. For each time point, tracer uptake ratios of the H/M ratio were calculated and defined as early H/M (30 min) and delayed H/M (120 min). The lower limits of the reference value for early and delayed H/M ratios were set to be 1.70 and 1.78, respectively^30^.

### Magnetic resonance imaging acquisition and cortical thickness measurements

The cortical thickness of 82 patients was analyzed by brain magnetic resonance imaging (MRI). A 3D T1-weighted magnetization-prepared rapid gradient-echo sequence was acquired using a 3-T scanner (Magnetom Verio, Siemens Healthcare, Erlangen, Germany) with a 12-channel head coil. Parameters were as follows: sagittal acquisition with FOV = 256 × 256 mm^2^; voxel size = 1 × 1 × 1 mm^3^; TR = 1780 msec; TE = 2.2 msec; flip angle = 9°; total acquisition time = 6 min 38 s. For the measurement of cortical thickness, we used the CIVET pipeline (http://mcin.ca/civet/) as described in detail elsewhere^39,40^. The native T1-weighted images of each subject were corrected for intensity inhomogeneity and spatially normalized to the MNI-152 template. After that, the images were tissue classified and hemispheric inner and outer cortical surfaces were automatically extracted using the constrained Laplacian-based automated segmentation with the proximities algorithm. Cortical thickness was measured by calculating the Euclidean distance between corresponding vertices on the gray matter/cerebrospinal fluid intersection surface and the white matter/gray matter boundary surface^41^. Diffusion smoothing with a 30-mm full width at half maximum kernel (FWHM) was used to increase the signal-to-noise ratio. To extract the mean regional cortical thickness, we used the Automated Anatomical Labeling (AAL) atlas template to define regional boundaries and averaged the cortical thickness of vertices within each of the regions of interest for each subject. Less than one third of the study population was included due to alterations of acquisition protocol and MRI equipment, and only patients with suitable uniformity for analyses were selected.

### Statistical analysis

All statistical analyses were performed with jamovi software (version 1.2.16; retrieved from https://www.jamovi.org) and R software with additional *car* and *emmeans* packages (version 3.6.3; retrieved from https://cran.r-project.org) for Mac. Descriptive analyses and the analysis of variance or Kruskal–Wallis tests were applied to describe the baseline characteristics of PD patients. Categorical variables were analyzed by the *χ*^2^ test. Subgroups of PD patients were examined by analysis of covariance, adjusted for age, sex, disease duration, and additional covariates when needed, to investigate between-group differences. To discern any linear gradient in the order of no-OH, delayed OH, and classical OH, polynomial contrasts or Cochran-Armitage tests were used, as appropriate. In a sub-group analysis of PD patients, repeated measures analysis of variance was applied to measure the temporal patterns of PD subgroups. Multiple comparisons were corrected with a defined significance at a two-tailed *p*-value < 0.05.

### Reporting summary

Further information on research design is available in the Nature Research Reporting Summary linked to this article.

## Supplementary information

Supplementary information

Reporting Summary

## Data Availability

Anonymized data generated during the current study are available from the corresponding author on reasonable request from individuals affiliated with research or health care institutions.

## Code availability

Jamovi is a statistical spreadsheet and graphical user interface (GUI) for R. All the analyses, except Cochran-Armitage, were performed basically using jamovi software as mentioned in the method, statistical analysis section. The R packages mentioned were utilized within jamovi GUI.
