# Supplementary material for: Delayed orthostatic hypotension in Parkinson’s disease
Source: NPJ Parkinsons Dis. 2021 Apr 14;7:37. doi: 10.1038/s41531-021-00181-y (PMC8047032; doi:10.1038/s41531-021-00181-y)
Supplement: Supplementary file 1 — Supplementary information [file 41531_2021_181_MOESM1_ESM.pdf]

Supplementary Table 1. Coefficients of linear polynomial contrasts

	Estimate	SE	t	P-value		Estimate	SE	t	P-value
UPDRS, total	3.55	1.15	3.08	0.002	Frontal	-0.08	0.02	-3.98	<0.001
UPDRS Part I	0.29	0.13	2.19	0.030	Right	-0.09	0.02	-3.83	<0.001
UPDRS Part II	1.14	0.39	2.95	0.003	Left	-0.08	0.02	-3.74	<0.001
UPDRS Part III	2.11	0.85	2.49	0.013	Parietal	-0.13	0.04	-3.19	0.002
MMSE	-0.381	0.26	-1.48	0.139	Right	-0.13	0.04	-3.02	0.004
Supine SBP	5.43	1.61	3.37	0.001	Left	-0.14	0.05	-3.07	0.003
Supine DBP	1.63	0.94	1.73	0.085	Temporal	-0.08	0.03	-2.43	0.018
Orthostatic ΔSBP	14.8	0.8	18.6	<0.0001	Right	-0.10	0.04	-2.71	0.008
Orthostatic ΔDBP	8.1	0.5	15.7	<0.0001	Left	-0.06	0.03	-1.78	0.079
Early H/M ratio	-0.12	0.03	-4.14	<0.0001	Occipital	-0.08	0.05	-1.83	0.072
Delayed H/M ratio	-0.15	0.04	-4.35	<0.0001	Right	-0.10	0.06	-1.81	0.074
NMSS total	11.96	4.63	2.58	0.011	Left	-0.07	0.05	-1.39	0.168
PDQ39 SI	1.85	1.42	1.30	0.194	Whole	-0.09	0.02	-3.59	0.001
OHQ Part I	1.94	1.04	1.86	0.065	Right	-0.10	0.03	-3.60	0.001
OHQ Part II	2.84	1.13	2.52	0.013	Left	-0.08	0.02	-3.19	0.002
MADRS sum	1.66	0.83	1.99	0.048					
RBDSQ	0.87	0.34	2.56	0.011					

Abbreviations: UPDRS, Unified Parkinson's Disease Rating Scale; MMSE, Mini-Mental Status Examination; SBP, systolic blood pressure; DBP, diastolic blood pressure; H/M, heart-to-mediastinum; NMSS, Non-Motor Symptoms Scale; PDQ39 SI, Parkinson's Disease Quality of Life-39 summary index; OHQ, Orthostatic Hypotension Questionnaire; MADRS, Montgomery-Asberg depression rating scale, RBDSQ, REM Sleep Behavior Disorder Screening Questionnaire; SE, Standard error

Supplementary Table 2. Influence of initial orthostatic subtypes on global cognitive efficiency and disease severity progression

	no-OH[a] (n = 27)		Delayed OH[b] (n = 19)		Classical OH[c] (n = 31)					
	Baseline	Follow-up	Baseline	Follow-up	Baseline	Follow-up	Time effect (F, P-values)	Group effect (F, P-values)	Post hoc analysis	Interaction (F, P-values)
UPDRS, total	24.4 ± 3.4	24.9 ± 3.4	24.3 ± 3.6	26.8 ± 3.6	30.4 ± 3.0	33.9 ± 3.0	0.56, 0.457	4.67, 0.012	a<c*	0.56 0.574
UPDRS Part I	1.2 ± 0.4	1.3 ± 0.4	1.5 ± 0.4	1.6 ± 0.4	1.4 ± 0.4	2.3 ± 0.4	0.18, 0.671	1.73, 0.184		2.03, 0.139
UPDRS Part II	6.9 ± 1.1	8.4 ± 1.1	7.2 ± 1.2	8.1 ± 1.2	9.2 ±1.0	10.5 ± 1.0	0.47, 0.495	3.99, 0.023	a<c*	0.14, 0.872
UPDRS Part III	16.3 ± 2.5	15.2 ± 2.5	15.5 ± 2.6	17.2 ± 2.6	19.8 ± 2.1	21.2 ± 2.1	0.60, 0.443	3.53, 0.034	a<c*	1.08, 0.346
MMSE	28.7 ± 0.8	27.9 ± 0.8	27.4 ± 0.8	26.7 ± 0.8	26.5 ± 0.7	26.0 ± 0.7	0.04, 0.840	5.54, 0.006	a>c**	0.14, 0.874

Abbreviations: OH, orthostatic hypotension; UPDRS, Unified Parkinson's Disease Rating Scale; MMSE, Mini-Mental Status Examination; CDR, Clinical dementia rating

Values are mean ± standard error of the mean unless otherwise indicated. Time, group, and time × group interaction effect values are P values.

Repeated measures analysis of covariance, adjusted for LEDD, was applied to discern within and between-group differences. Pairwise multiple comparisons were adjusted by Tukey's HSD.

* p-value <0.05, ** p-value <0.01, *** p-value <0.001

Supplementary Figure 1. Temporal progression of cognitive and motor severities across the groups, adjusted of dopaminergic replacement therapy

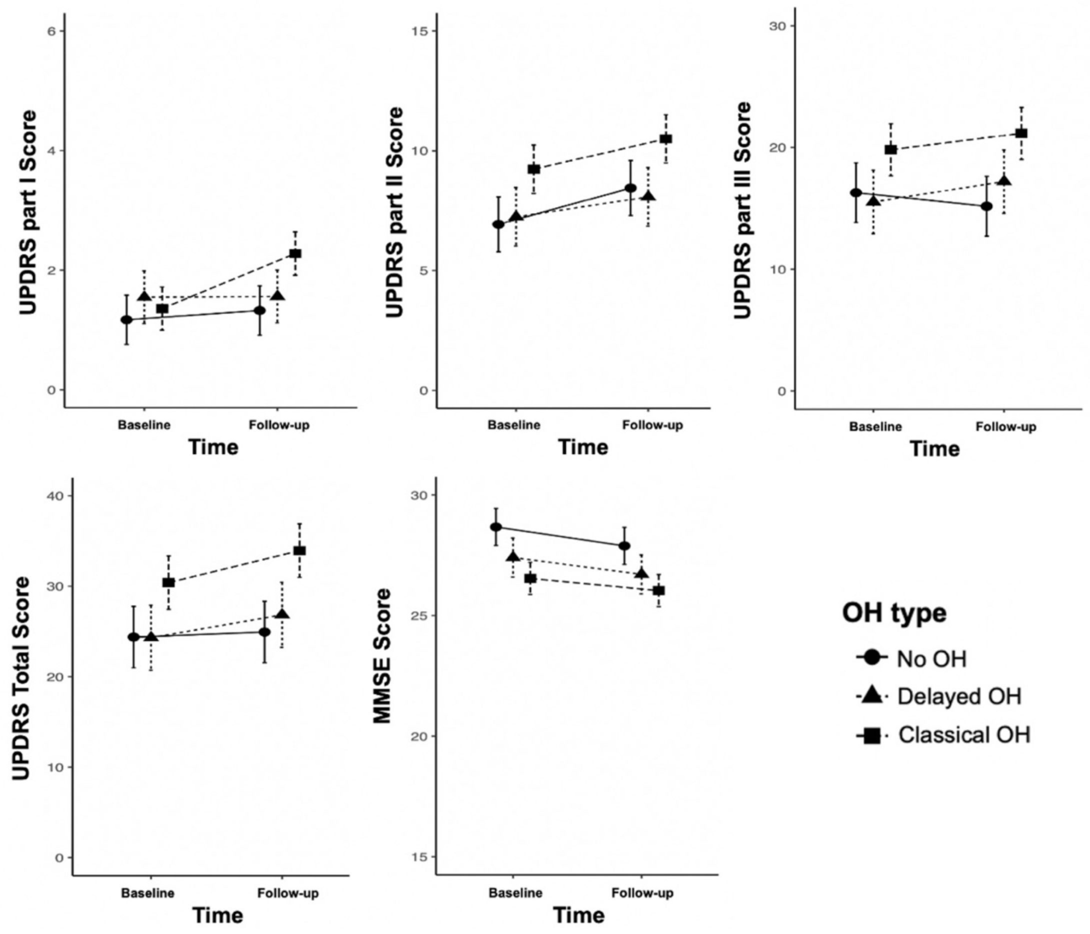

Error bars: standard error of the mean

Abbreviations: UPDRS, Unified Parkinson's Disease Rating Scale; MMSE, Mini-Mental Status Examination; OH, orthostatic hypotension
