# Supplementary material for: Delayed orthostatic hypotension in Parkinson’s disease
Source: NPJ Parkinsons Dis. 2021 Apr 14;7:37. doi: 10.1038/s41531-021-00181-y (PMC8047032; doi:10.1038/s41531-021-00181-y)
Supplement: Supplementary file 2 — Reporting Summary [file 41531_2021_181_MOESM2_ESM.pdf]

nature research

Corresponding author(s): NPJPARKD-00515R

Last updated by author(s): Jan 11, 2021

Reporting Summary

Nature Research wishes to improve the reproducibility of the work that we publish. This form provides structure for consistency and transparency in reporting. For further information on Nature Research policies, see our Editorial Policies and the Editorial Policy Checklist.

Statistics

For all statistical analyses, confirm that the following items are present in the figure legend, table legend, main text, or Methods section.

n/a	Confirmed	
☐	☒	The exact sample size (n) for each experimental group/condition, given as a discrete number and unit of measurement
☐	☒	A statement on whether measurements were taken from distinct samples or whether the same sample was measured repeatedly
☐	☒	The statistical test(s) used AND whether they are one- or two-sided<br>*Only common tests should be described solely by name; describe more complex techniques in the Methods section.*
☐	☒	A description of all covariates tested
☐	☒	A description of any assumptions or corrections, such as tests of normality and adjustment for multiple comparisons
☐	☒	A full description of the statistical parameters including central tendency (e.g. means) or other basic estimates (e.g. regression coefficient) AND variation (e.g. standard deviation) or associated estimates of uncertainty (e.g. confidence intervals)
☐	☒	For null hypothesis testing, the test statistic (e.g. F, t, r) with confidence intervals, effect sizes, degrees of freedom and P value noted<br>*Give P values as exact values whenever suitable.*
☒	☐	For Bayesian analysis, information on the choice of priors and Markov chain Monte Carlo settings
☒	☐	For hierarchical and complex designs, identification of the appropriate level for tests and full reporting of outcomes
☒	☐	Estimates of effect sizes (e.g. Cohen's d, Pearson's r), indicating how they were calculated

Our web collection on statistics for biologists contains articles on many of the points above.

Software and code

Policy information about availability of computer code

Data collection	Data collection was done by using Microsoft Excel. The files were then converted into csv files for statistical programs.
Data analysis	For data analysis, jamovi software (version 1.2) and R software (version 3.6) were used. Customized codes were not used.

For manuscripts utilizing custom algorithms or software that are central to the research but not yet described in published literature, software must be made available to editors and reviewers. We strongly encourage code deposition in a community repository (e.g. GitHub). See the Nature Research guidelines for submitting code & software for further information.

Data

Policy information about availability of data

All manuscripts must include a data availability statement. This statement should provide the following information, where applicable:

- Accession codes, unique identifiers, or web links for publicly available datasets
- A list of figures that have associated raw data
- A description of any restrictions on data availability

Anonymized data generated during the current study are available from the corresponding author on reasonable request from individuals affiliated with research or health care institutions.

Field-specific reporting

Behavioural & social sciences study design

All studies must disclose on these points even when the disclosure is negative.

Study description	This is a longitudinal observational study with quantitative data.
Research sample	Research sample is selected among patients who visit our institution's movement clinic at Seoul St Mary's hospital. The mean age was 69.9 ± 9.3 years old and 132 (46.3%) were female. The sample is representative of PD population of a single center. It is from our cohort registry that collects data retrospectively and prospectively. Participants with analyzable data were selected from this registry, so the sample size was not calculated.
Sampling strategy	There was no sample-size calculation. This was an observatory study from cohort registry that collects data. But to ensure normality in the analysis at least 30 participants were thought to be required for each group. Furthermore, as PD patients are frequently lost during the follow-up period, at least 200 patients were thought to be required for analyses.
Data collection	Data collection was done by pen and paper and computer as appropriate. During surveys of questionnaires, an experienced personnel was present by side. The researcher was not blind to experimental condition as it was not required in this study.
Timing	Data started at October 2014 and ended at December 2019.
Data exclusions	Among 285 enrolled participants, 177 of them went through surveys of questionnaires. Those who did not complete them were excluded from the sub-analyses. 82 participants were available for MRI interpretation. Due to changes in MRI protocol, only those whose MRI parameters were suitable for analyses were selected. 77 people were followed of their disease severity, and many of the initially enrolled participants were lost during the follow-up period.
Non-participation	Of 285 participants, 208 were dropped out because they were lost during the follow-up.
Randomization	Participants were not allocated into experimental groups.

Reporting for specific materials, systems and methods

We require information from authors about some types of materials, experimental systems and methods used in many studies. Here, indicate whether each material, system or method listed is relevant to your study. If you are not sure if a list item applies to your research, read the appropriate section before selecting a response.

Materials & experimental systems

n/a	Involved in the study	
☒	☐	Antibodies
☒	☐	Eukaryotic cell lines
☒	☐	Palaeontology and archaeology
☒	☐	Animals and other organisms
☐	☒	Human research participants
☒	☐	Clinical data
☒	☐	Dual use research of concern

Methods

n/a	Involved in the study	
☒	☐	ChIP-seq
☒	☐	Flow cytometry
☒	☐	MRI-based neuroimaging

Human research participants

Policy information about studies involving human research participants

Population characteristics	See above
Recruitment	Participants who visited our movement clinic were recruited with their consent. As this study enrolled at a single center, this may cause selection bias.
Ethics oversight	This study was approved by the Institutional Review Board at Seoul St. Mary's Hospital.

Note that full information on the approval of the study protocol must also be provided in the manuscript.
